# Can a TB outbreak be detected by reviewing the surveillance data?

**DOI:** 10.5588/pha.25.0026

**Published:** 2025-12-03

**Authors:** R. Yamaguchi, T. Umezawa, N. Date, W. Furusawa, T. Maeki, K. Uchimura, S. Hirao, M. Ota

**Affiliations:** 1 Sapporo City Health Office, Sapporo, Japan;; 2 Research Institute of Tuberculosis, Japan Anti-Tuberculosis Association, Tokyo, Japan.

**Keywords:** tuberculosis, Sapporo, Japan, disease outbreaks, epidemiology

## Abstract

**OBJECTIVE:**

To retrospectively review TB surveillance data to detect whether there was a possible outbreak in the area and verify it in the city of Sapporo, a megalopolis with a low burden of TB.

**DESIGN:**

A cohort study in which TB notification rates of wards each year were compared with those for the rest of the city. If the rate was significantly higher, the notification rates by sex and age groups were further compared with those of the rest of the city.

**RESULTS:**

Six possible TB outbreaks were found in six wards: Chuo in 2007, Atsubetsu in 2010, Higashi in 2014, Shiroishi in 2015, Nishi in 2017, and Minami in 2018. Further analysis found that the notification rates were significantly higher in specific sex and age groups than those of the rest of the city. The city’s outbreak records showed three actual outbreaks (one in Atsubetsu ward in 2010 and two in Higashi in 2014) corresponding to two events found in our analysis.

**CONCLUSION:**

Our study shows that retrospectively reviewing TB surveillance data could detect possible outbreaks. Local health offices and prefectures of Japan should monitor their TB surveillance data at least monthly to detect possible outbreaks and take appropriate actions if needed.

Public health surveillance is the continuous, systematic collection, analysis, and interpretation of health-related data.^1^ Disease surveillance data serve as an early warning system for impending outbreaks that could become public health emergencies.^1^ Public health surveillance systems in various countries have detected outbreaks of Guillain–Barré syndrome, enterohaemorrhagic *Escherichia coli* O157, cholera, leptospirosis, and typhoid fever.^2–6^ For TB, we previously reported a diffused outbreak in Japan that might have involved patrons of a casino and that could have been detected earlier by continuously reviewing the surveillance data.^7^ The United States Centers for Disease Control and Prevention (US-CDC) monitored TB surveillance data and detected a resurgence of TB in late 1985.^8^ Also, in the United States, outbreaks were retrospectively detected using the national TB surveillance data and genotyping.^9,10^ However, there are only a limited number of reports in relation to detection of possible TB outbreaks using the surveillance data in Japan and other countries.

In Japan, the TB notification rate has declined 86 fold in the past seven decades from 698 per 100,000 population in 1951 to 8.1 per 100,000 population in 2023.^11,12^ However, about 4,000 smear-positive TB cases are still reported every year,^11^ and these infectious TB cases pose a public health threat to the community.^13–15^ TB outbreaks involving hospitals, workplaces, schools, military exercises, and homeless persons have also been reported.^16–25^ As the TB notification rates decline, it is possible that TB outbreaks could more easily be detected by continuously prospectively monitoring and/or retrospectively reviewing TB surveillance data. In this study, we retrospectively reviewed the TB surveillance data of the city of Sapporo for the 17 years from 2007 through 2023 to see whether we could detect possible outbreaks and verify whether there were actual ones.

## METHODS

The city of Sapporo, with an area of 1,121 km^2^, is located in the western part of Hokkaido, the northernmost main island of Japan.^26^ The city is comprised of ten districts: Chuo, Kita, Higashi, Shiroishi, Toyohira, Minami, Nishi, Atsubetsu, Teine, and Kiyota wards. The city’s population was 1.96 million in 2023, 28.5% of whom were aged 65 years or older. Its population has increased by 4.3% from 1.88 million in 2005.^26^

In this study, a TB case was defined as one in which TB was diagnosed based on a bacteriologically positive sputum sample by smear microscopy, culture, or nucleic acid amplification, or by a physician via a chest X-ray and/or computed tomography, histological or pathological tests, or clinically registered in the city of Sapporo from 2007 through 2023. A TB outbreak was defined as an event in which the number of cases of TB reported in a particular area within a particular time frame (normally 1 year) was significantly higher than in surrounding areas. All the cases reported to the surveillance system were included in the study, and none of the cases was excluded.

Epidemiological information on patients with TB in the study area was extracted from the national surveillance data for cases of TB registered from 2007 through 2023, including sex, age, the ward of residence, and type of TB disease (either pulmonary or extra-pulmonary). No patients’ names were included in the national TB surveillance data.

First, the TB notification rates of a ward were compared with those of the rest of the entire city of Sapporo by year by calculating relative risks (RRs) of the former compared to the latter. When an RR for a ward compared to that for the rest of the city in a specific year was significantly higher than 1.0, we further reviewed RRs of the ward compared to those for the rest of the city in that year by sex and by 10-year-interval age groups as mentioned above. We also reviewed the records in relation to TB outbreaks documented by the city health office. Since the city started molecular epidemiological studies from 2011 onward, we also reviewed the variable number of tandem repeat (VNTR) results in and around that specific year, if available.

Statistical tests were carried out with R (Ver. 4.0.2., The R Foundation for Statistical Computing, Vienna, Austria). Fisher’s exact test was conducted for calculating RRs. Ninety-five per cent confidence intervals (CIs) were calculated assuming that the notification rates for cases of TB had a binomial distribution. Fisher’s exact test was employed for comparison of proportions. A *P* value of less than 0.05 was considered statistically significant.

A waiver for the ethical review for the study was obtained from the Institutional Review Board of the Research Institute of Tuberculosis (Decision letter # #2025-08 of RIT-IRB).

## RESULTS

From 2007 through 2023, 2,892 cases of TB were reported in the city of Sapporo with an average of 170.1 cases per year. The notification rates of TB decreased by 63.8% in the city from 13.0 per 100,000 in 2007 to 4.7 per 100,000 in 2023.

**FIGURE 1.**
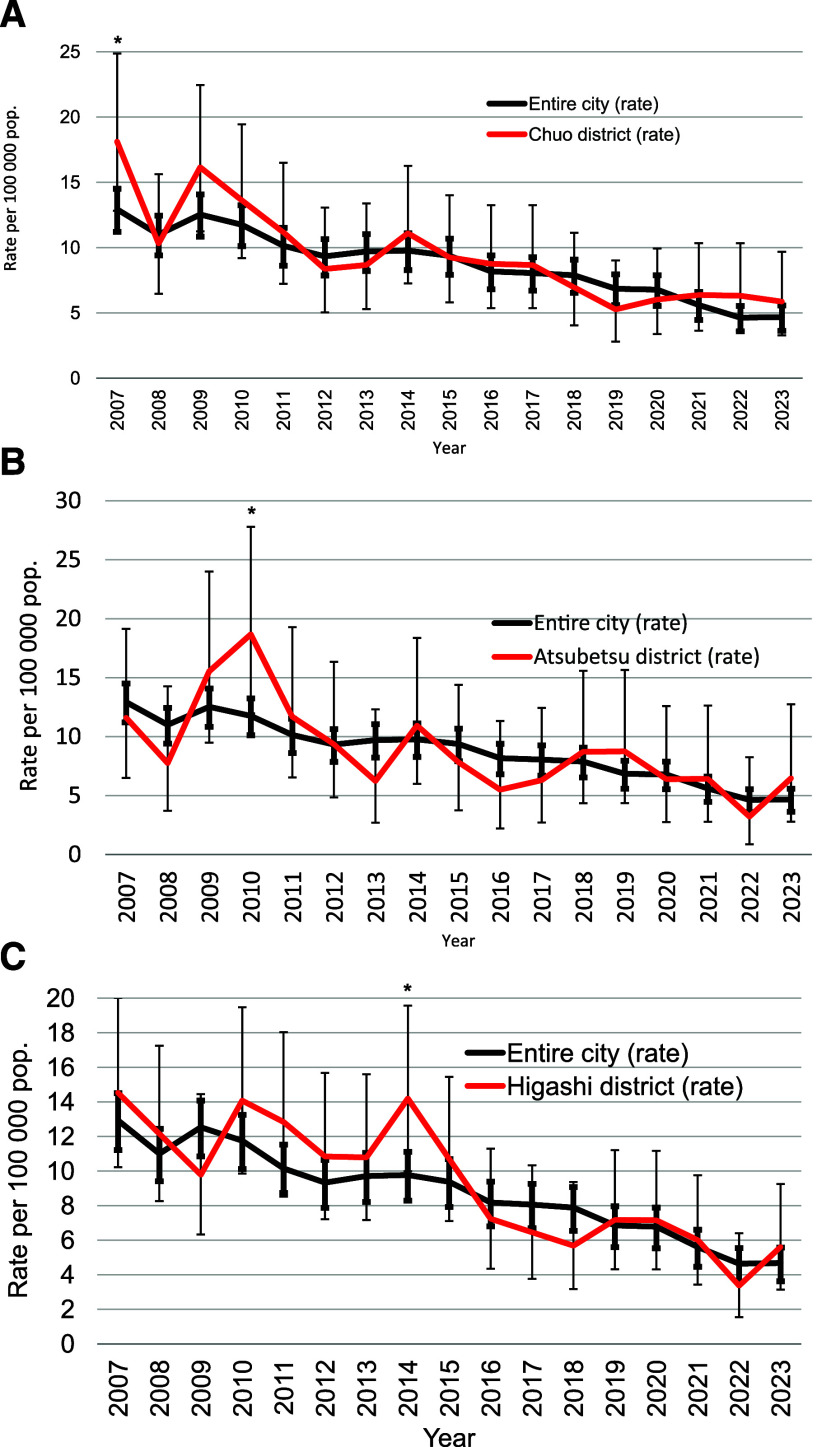

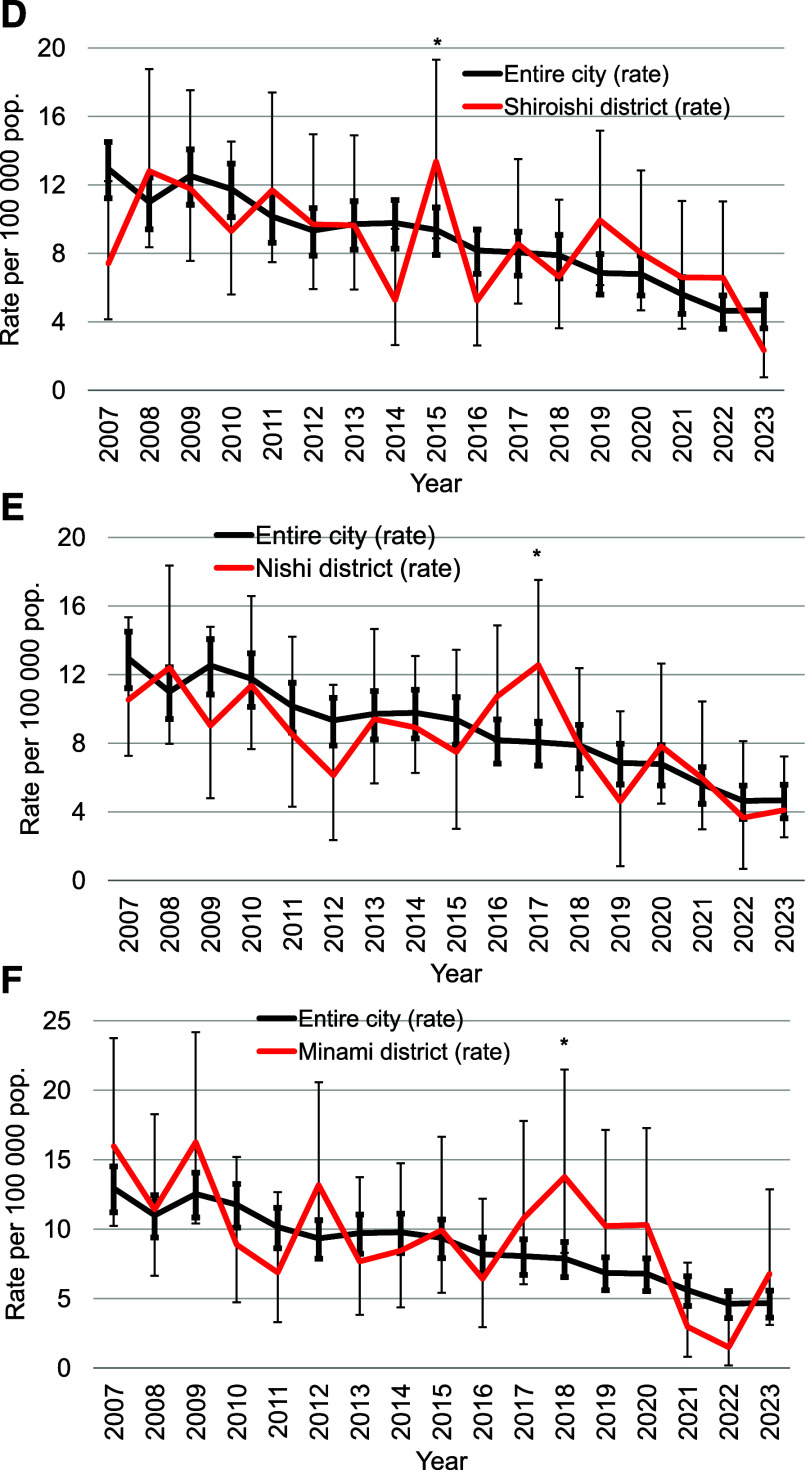
TB notification rates of the entire city of Sapporo and the wards in which possible diffused outbreaks were detected, Sapporo, Japan, 2007–2023. **A)** Chuo ward; **B)** Atsubetsu ward; **C)** Higashi ward; **D)** Shiroishi ward; **E)** Nishi ward; **F)** Minami ward. Bars indicate 95% confidence intervals. An asterisk indicates that in a specific year the notification rate of the ward shown was significantly higher than for the rest of the city. pop. = population.

**FIGURE 2.**
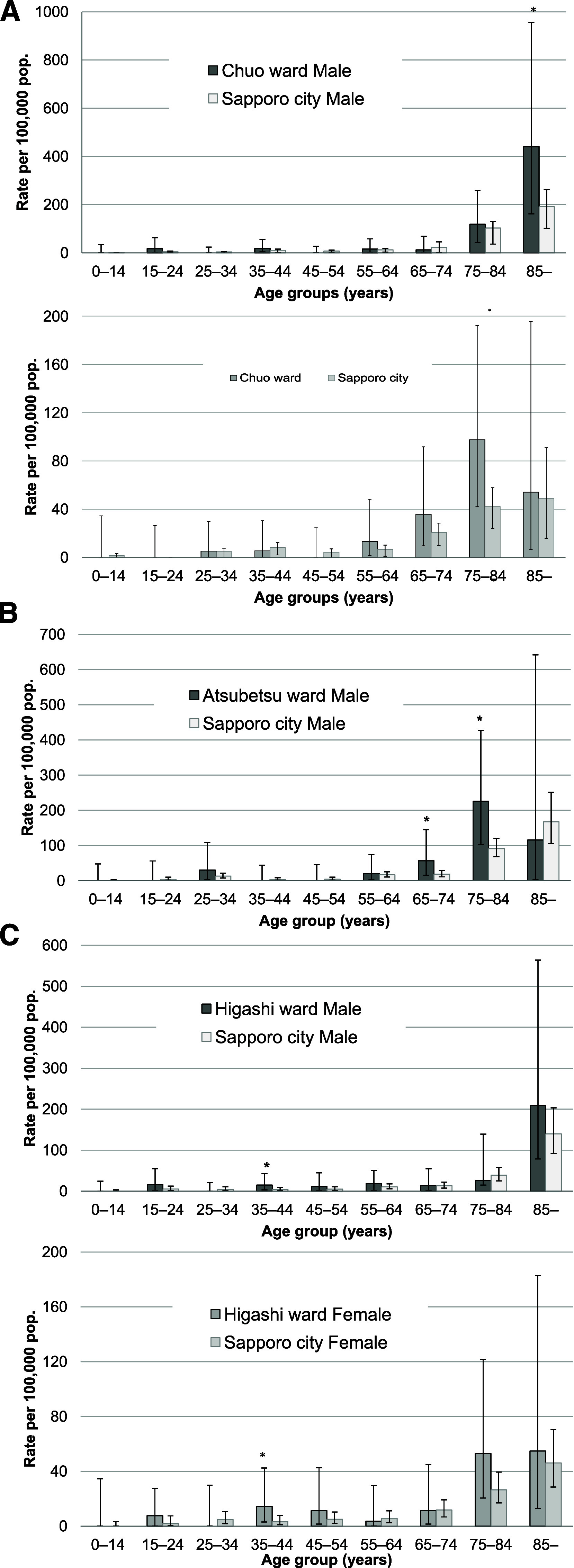

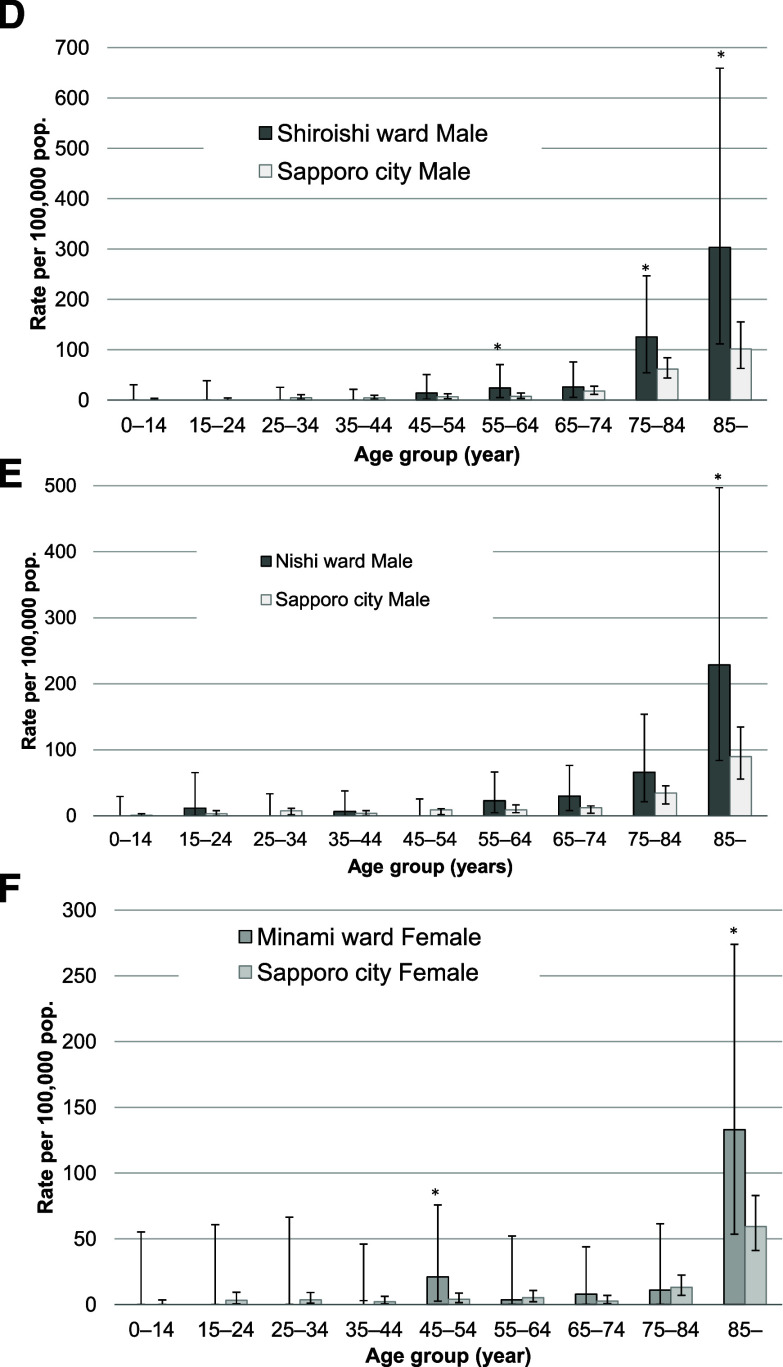
Age- and sex-specific TB notification rates of the entire city of Sapporo and the wards in which possible outbreaks were detected, Sapporo, Japan, 2007–2023. **A)** Male (upper) and female (lower) notification rates of TB in Chuo ward in 2007; **B)** male notifications rates of TB in Atsubetsu ward in 2010; **C)** male (upper) and female (lower) notification rates of TB in Higashi ward in 2014; **D)** male notification rates of TB in Shiroishi ward in 2015; **E)** male notification rates of TB in Nishi ward in 2017; **F)** female notification rates of TB in Minami ward in 2018. Bars indicate 95% confidence intervals. An asterisk indicates that in a specific sex and age group the notification rate of the ward was significantly higher than for the rest of the city (see the main text). pop. = population.

During the study period, we retrospectively detected six possible TB outbreaks in six wards of the city of Sapporo (Table): in Chuo in 2007 (RR: 1.5, 95% CI: 1.0–2.1), Atsubetsu in 2010 (RR: 1.6, 95% CI: 1.1–2.3), Higashi in 2014 (RR: 1.5, 95% CI: 1.1–1.9), Shiroishi in 2015 (RR: 1.4, 95% CI: 1.0–2.0), Nishi in 2017 (RR: 1.6, 95% CI: 1.1–2.2), and Minami in 2018 (RR: 1.7, 95% CI: 1.1–2.7), because the RRs of TB notification rates of the wards compared to those for the rest of the city were significantly higher. Figure 1A–F shows the TB notification rates of the wards compared with that of the entire city from 2007 through 2023. We further calculated RRs of the notification rates of TB in the six wards in those years by sex and age groups to those in the rest of the city and found that the RRs of TB in specific sex and age groups were statistically significant in 1) males over 85 years old (RR: 2.8, 95% CI: 1.1–7.1) and 75–84-year-old females (RR: 2.8, 95 %CI: 1.2–6.2) in Chuo ward in 2007, 2) 65–74 (RR: 3.7, 95% CI: 1.2–11.5) and 75–84-year-old males (RR: 2.8, 95% CI: 1.4–5.7) in Atsubetsu ward in 2010, 3) 35–44-year-old males (RR: 5.9, 95% CI: 1.2–29.2) and females (RR: 9.4, 95% CI: 1.6–56.5) in Higashi ward in 2014, 4) 55–64 (RR: 4.4, 95% CI: 1.1–17.7), 75–84 (RR: 2.3, 95% CI: 1.1–5.0), and over-85-year-old (RR: 3.8, 95% CI: 1.5–9.7) males in Shiroishi ward in 2015, 5) over-85-year-old (RR: 3.2, 95% CI: 1.2–8.2) males in Nishi ward in 2017, and 6) 45–54 (RR: 7.3, 95% CI: 1.3–39.9) and over-85-year old (RR: 2.6, 95% CI: 1.1–5.9) females in Minami ward in 2018 (Table and Figure 2).

**TABLE.** Summary of the findings in relation to the detection of the possible outbreaks from the surveillance data and the actual outbreak records of the city health office, Sapporo, 2007–2023.

Year	Ward	RR of the notification rate of the ward to the rest of the city	Sex and age group in which the notification rates were higher than that of the rest of the city	Corresponding actual outbreak(s) recorded
2007	Chuo	1.5, 95% CI: 1.0–2.1	Males aged >85 years (RR: 2.8, 95% CI: 1.1–7.1) and females aged 75–84 years (RR: 2.8, 95% CI: 1.2–6.2).	Not recorded.
2010	Atsubetsu	1.6, 95% CI: 1.1–2.3	Males aged 65–74 (RR: 3.7, 95% CI: 1.2–11.5) and 75–84 years (RR: 2.8, 95% CI: 1.4–5.7).	Nosocomial outbreak in a hospital in which altogether 10 patients were found between 2008 and 2010.
2014	Higashi	1.5, 95% CI: 1.1–1.9	Males (RR: 5.9, 95% CI: 1.2–29.2) and females (9.4, 95% CI: 1.6–56.5) both aged 35–44 years.	Three male patients were found in a prison in late 2014 and early 2015.
				Four male and female patients were found in a factory from late 2014 to early 2015.
2015	Shiroishi	1.4, 95% CI: 1.0–2.0	Males aged 55–64 (RR: 4.4, 95% CI: 1.1–17.7), 75–84 (RR: 2.3, 95% CI: 1.1–5.0), and >85 years (RR: 3.8, 95% CI: 1.5–9.7).	Not recorded.
2017	Nishi	1.6, 95% CI: 1.1–2.2	Males aged >85 years (RR: 3.2, 95% CI: 1.2–8.2).	Not recorded.
2018	Minami	1.7, 95% CI: 1.1–2.7	Females aged 45–54 (RR: 7.3, 95% CI: 1.3–39.9) and >85 years (RR: 2.6, 95% CI: 1.1–5.9).	Not recorded.

CI = confidence interval; RR = risk ratio.

We also reviewed the records in relation to TB outbreaks that occurred in the city of Sapporo and found that there were three TB outbreaks matching the above-mentioned events of the possible outbreaks by time and place: one in a prison and another in a factory, both in Higashi ward in 2014, in which three patients with active TB were found in the former and four patients were found in the factory. Another outbreak occurred in a hospital in Atsubetsu ward in 2008–2010, in which altogether 10 patients with active TB were found (Table). In addition, we reviewed the available analysis based on the VNTR for the TB bacilli collected from patients with TB after 2011. We were not able to find any clusters of patients with TB from the VNTR analysis in relation to the possible diffused TB outbreaks among Chuo ward in 2007, Shiroishi ward in 2015, Nishi ward in 2017, and Minami ward in 2018.

## DISCUSSION

We retrospectively reviewed the national TB surveillance data from the city of Sapporo from 2007 through 2023 and found six possible TB outbreaks in six wards, of which two events corresponded to three actual facility-based outbreaks: one in a prison and another in a factory in Higashi ward in 2014, and the other in a hospital in Atsubetsu ward in 2008–2010 from the records of the city health office; however, it was not possible to verify the other four, even using VNTR analysis.

Public surveillance systems have been shown to be effective in detecting various disease outbreaks,^3–9^ including a diffused TB outbreak in Japan, in which we retrospectively detected a possible TB outbreak by analysing notification rates as a whole and by age group.^7^ Our study has added one more example in which TB outbreaks could retrospectively, and thus also prospectively, be detected by monitoring TB surveillance data. In Japan, the number of cases with TB has decreased and it has become easier to detect small TB outbreaks with as few as 7–10 cases of active TB, as shown in this study.

This study is the second report on the practice of reviewing TB surveillance data retrospectively, showing that it is possible to detect diffused TB outbreaks. However, there are also a few limitations. First, we conducted an epidemiological review of the national TB surveillance data but did not conduct a field investigation, except for one outbreak in Atsubetsu ward in 2010 and two in Higashi ward in 2014, as mentioned in the Results section. Therefore, the possible TB outbreaks detected with our method may represent false-positive results. Second, molecular epidemiological investigation was not comprehensively conducted because the practice started in 2011, and thus one of the possible diffused outbreaks that occurred before 2011 in Chuo ward were not investigated. It should also be noted that about one sixth of the patients with pulmonary TB were culture-negative^27^ and VNTR analysis was not possible for their bacilli. VNTR analysis and/or whole genome sequencing are not perfect tools, and epidemiology and molecular biology should go hand in hand to tackle diffused TB outbreaks. Third, this study is the second example of trying to detect diffused community TB outbreaks, and the findings and the lessons learnt might not be generalisable to other settings; however, the authors would like to emphasise that it is of importance to countries with a low to a medium burden of TB in terms of utilisation of surveillance data and an early warning system.

We recommend that local health offices and prefectures in Japan and, perhaps, countries with a low to medium burden of TB should monitor their TB surveillance data by medium administrative area (with 100,000–200,000 population) at least on a monthly basis and initiate an outbreak investigation if the case rate of TB patients reported exceeds the rate of the surrounding area.
